# P-326. Where Does the Glove Fit? Examining the Effect of Hand Hygiene Timing on Healthcare Personnel Glove Contamination

**DOI:** 10.1093/ofid/ofae631.529

**Published:** 2025-01-29

**Authors:** Erin Gettler, Bobby G Warren, Guerbine Fils-Aime, Aaron Barrett, Amanda M Graves, Deverick J Anderson, Becky A Smith

**Affiliations:** Duke University Medical Center, Durham, NC; Duke University School of Medicine, Hillsborough, North Carolina; Duke School of Medicine, Durham, North Carolina; Duke Health, Cary, North Carolina; Duke University School of Medicine Duke Center for Antimicrobial Stewardship and Infection Prevention, Durham, North Carolina; Duke Center for Antimicrobial Stewardship and Infection Prevention, Durham, NC; Duke University, durham, North Carolina

## Abstract

**Background:**

Guidelines recommend performing hand hygiene (HH) prior to donning non-sterile gloves, yet strong evidence is lacking and overall adherence is low. This study evaluated the impact of different HH and gloving strategies on glove contamination.

**Figure:**
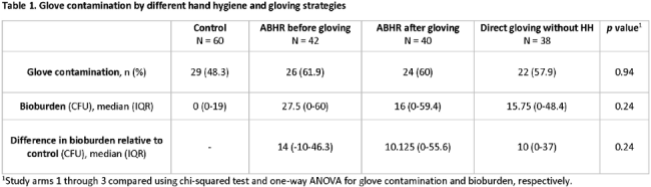

**Methods:**

Healthcare personnel (HCP) were enrolled on inpatient units and then randomized into 3 arms: 1) standard practice of alcohol-based hand rub (AHBR) before gloving, 2) ABHR after gloving, or 3) direct gloving without HH. Study personnel first collected 1 control glove per HCP from the same glove box used after randomization. After donning, HCP gloves were aseptically removed and placed into sterile bags by study personnel (left, right, control). Inverted gloves were aseptically filled with 50 mL of neutralizing buffer, sealed, and agitated. Buffer was centrifuged at 3100 rpm for 15 minutes and decanted leaving only ∼3 mL of sample. Each homogenate was vortexed and plated onto routine media to assess for bioburden and epidemiologically important pathogens (EIP), including *Staphylococcus aureus*, *Enterococcus* species, and gram-negative bacteria. Gloves were visually inspected and tested for microperforations by the water inflation test. Rate of glove contamination and bioburden were compared.

**Results:**

60 HCP across 8 inpatient units were enrolled. Control gloves obtained were frequently contaminated (48.3%). Compared to standard practice, neither HH after gloving nor direct gloving led to significant differences in glove contamination or bioburden (Table 1). In fact, HH after gloving trended toward lower bioburden on the gloves, but this result was not significant. Importantly, the application of ABHR to gloves did not compromise the integrity of the glove or result in microperforations. Gloves were relatively void of EIP.

**Conclusion:**

In this exploratory analysis, HH after donning non-sterile gloves or direct gloving did not result in higher glove contamination. These techniques may represent safe alternative HH practices for HCP and circumvent some of the common barriers limiting HH compliance. Additional studies are needed.

**Disclosures:**

**Becky A. Smith, MD**, UpToDate: royalties

